# Promising Molecular Therapeutic Targets for Drug Development in Rheumatoid Arthritis

**DOI:** 10.3390/jcm14165827

**Published:** 2025-08-18

**Authors:** Sang Wan Chung

**Affiliations:** Division of Rheumatology, Department of Internal Medicine, College of Medicine, Kyung Hee University, Seoul 02453, Republic of Korea; wanyworld83@gmail.com

**Keywords:** rheumatic disease, molecular target, novel treatment

## Abstract

Rheumatic diseases encompass various autoimmune and inflammatory conditions affecting the joints, muscles, and connective tissues. Recent studies have advanced our understanding of the molecular pathogenesis of rheumatic diseases and have led to the identification of several promising therapeutic targets, paving the way for the development of novel drug interventions. This review highlights the latest molecular advances in rheumatoid arthritis, with a particular focus on innovative therapeutic strategies, and presents new perspectives on their management, including cytokines, intracellular signaling pathways, immune checkpoints, and novel cell-based therapies. A better understanding of these targets is essential for developing effective and personalized treatments.

## 1. Introduction

Rheumatic diseases, such as rheumatoid arthritis (RA), are chronic autoimmune disorders characterized by chronic inflammation and joint damage [1]. RA is a disease that can affect daily life by causing joint deformities if not diagnosed and treated early. Therefore, early diagnosis and appropriate treatment are important in RA. Current treatment guidelines recommend applying a treatment-to-target strategy, and using conventional, biological, and new non-biological disease-modifying antirheumatic drugs [2]. The complex pathogenesis of RA is reflected in the diversity and intricacy of molecular targets that can be selected for treatment. These include cytokines, cytokine receptors, kinases downstream of these receptors, cell-surface molecules that mediate immune interactions between various leukocyte populations, and receptors that can serve as targets for the depletion of pathogenic subsets of immune cells (Table 1). Despite advances in disease-modifying antirheumatic drugs and biologics, such as tumor necrosis factor (TNF) and Janus kinase (JAK) inhibitors, many patients with RA exhibit inadequate responses or develop resistance over time [3]. Consequently, identifying novel molecular targets is essential for developing more effective therapies. This review highlights emerging molecular targets in RA, focusing on key signaling pathways and potential therapeutic strategies.

## 2. Novel Molecular Targets (Table 1)

### 2.1. Bruton’s Tyrosine Kinase (BTK) Inhibitors

BTK is a non-receptor tyrosine kinase, a key component of the B cell receptor signaling pathway, and plays a critical role in B cell activation and autoantibody production [38]. BTK inhibition has shown promise in systemic lupus erythematosus (SLE) and RA, and molecules, such as fenebrutinib and evobrutinib, are currently under clinical investigation.

Fenebrutinib, a selective inhibitor of BTK, has shown promising efficacy in randomized trials involving RA patients with inadequate response to methotrexate (MTX). In a Phase II clinical trial, patients with RA who had an inadequate response to MTX were randomized to receive fenebrutinib, adalimumab, or placebo, and all patients continued MTX therapy. The primary endpoint of the trial was the American College of Rheumatology 50% (ACR50) response at week 12, while secondary endpoints included changes in the Disease Activity Score in 28 joints using C-reactive protein (DAS28-CRP), DAS28 using the erythrocyte sedimentation rate (DAS28-ESR), and the Health Assessment Questionnaire Disability Index (HAQ-DI). Patients who received high-dose fenebrutinib up to week 12 had significantly higher ACR50 responses than patients in the placebo group. Moreover, the efficacy of adalimumab and fenebrutinib was similar. In addition, fenebrutinib had a similar incidence of adverse events as adalimumab [4]. Given the safety and efficacy of fenebrutinib and adalimumab, there is potential for further development as additional clinical trials are conducted. In a Phase II trial, another BTK inhibitor, evobrutinib, did not show statistically significant improvement in ACR20 scores compared to placebo in patients with RA. However, a favorable safety profile was observed, along with improvements in DAS28-CRP scores [5].

### 2.2. Mitochondrial Complex I Inhibitor

Leramistat (MBS2320) is an investigational small molecule that targets mitochondrial complex I with preferential activity in osteoclasts, thereby offering a dual mechanism of anti-inflammatory and anti-resorptive action. Its capacity to modulate osteoclast metabolism positions it as a promising therapeutic candidate for the treatment of RA. Preclinical models have provided robust evidence of its efficacy in preserving bone architecture and attenuating inflammatory responses in arthritic joints [39].

A Phase II randomized, placebo-controlled trial was conducted to assess the clinical safety and efficacy of MBS2320 in patients with active, severe RA who were maintained on stable doses of MTX. The primary endpoint of the study was safety and tolerability over 12 weeks, which was successfully met with no drug-related serious adverse events. Secondary endpoints included clinical efficacy assessments such as ACR20 response and change in DAS28-CRP, as well as exploratory evaluations of inflammatory markers and imaging outcomes. The study revealed that patients treated with MBS2320 achieved consistently high ACR20 response rates at all evaluated time points compared to the placebo. Additionally, a significant improvement in disease activity was reflected in DAS28-CRP scores among individuals in the treatment group; however, DAS28-ESR was not analyzed in this study. The most frequently reported adverse events included nausea and other gastrointestinal symptoms, which were generally mild and well tolerated [6]. These findings support the further investigation of MBS2320 as a novel therapeutic agent with potential to address both inflammatory and osteolytic components of RA pathology.

### 2.3. NOD-, LRR-, and Pyrin Domain-Containing Protein 3 (NLRP3) Inflammasome Inhibitors

The NLRP3 inflammasome is a key intracellular sensor that contributes to innate immune activation by facilitating the maturation and release of interleukin (IL)-1β, a potent pro-inflammatory cytokine. Inflammasomes are multiprotein signaling platforms that detect pathogen-associated molecular patterns (PAMPs) and damage-associated molecular patterns (DAMPs) through pattern recognition receptors (PRRs). Canonically, the inflammasome complex consists of a sensor PRR, the adaptor protein ASC, and pro-caspase-1, which upon assembly leads to the proteolytic activation of caspase-1 [40]. This activation drives the processing and secretion of IL-1β and IL-18, both members of the IL-1 cytokine family, which are integral to the regulation of both innate and adaptive immunity [41].

The NLRP3 inflammasome has been extensively investigated in joint diseases where inflammasome activation is a central pathogenic mechanism, with initial translational evidence emerging in gout and osteoarthritis (OA). In gout, monosodium urate crystals potently activate NLRP3, driving IL-1β release and acute inflammation. The selective oral NLRP3 inhibitor dapansutrile (OLT1177) significantly reduced joint pain, swelling, and inflammatory markers in a Phase IIa randomized, placebo-controlled trial during acute flares, with a favorable safety profile [42]. In OA, low-grade chronic inflammation mediated by NLRP3 contributes to cartilage degradation and synovial inflammation. Preclinical studies using inhibitors such as MCC950 and DFV890 have demonstrated reduced cartilage loss and synovial inflammation, and DFV890 has entered early-phase clinical testing in patients with knee OA (NCT04886258) [43]. These findings provided proof-of-concept for the therapeutic potential of NLRP3 inhibition arthritis.

Among inflammasome subtypes, NLRP3 has garnered particular attention due to its pivotal role in mediating inflammation in RA. By linking innate and adaptive immune pathways, NLRP3 inflammasome activation triggers caspase-1-dependent processing of IL-1β and IL-18, thereby amplifying inflammatory signaling. IL-1β, in particular, is abundantly produced by monocytes, macrophages, and dendritic cells, and promotes leukocyte recruitment by inducing chemokines and adhesion molecules [44]. This pro-inflammatory cascade contributes not only to synovial inflammation but also to joint destruction through cartilage degradation and bone erosion [45].

Accumulating evidence implicates aberrant NLRP3 activation in RA pathogenesis. Anti-citrullinated protein antibodies (ACPAs), the hallmark biomarkers of RA, have been shown to stimulate NLRP3 inflammasome assembly and promote IL-1β secretion in immune cells derived from RA patients [7]. Furthermore, the elevated expression of NLRP3 and its associated components has been reported in monocytes and antigen-presenting cells from individuals with RA [8]. Given these insights, the pharmacologic inhibition of NLRP3 represents a promising therapeutic strategy. Compounds such as dapansutrile and other NLRP3-targeting small molecules are under investigation for their potential to modulate inflammasome-driven inflammation in RA and related auto-inflammatory disorders.

### 2.4. Regulatory T Cells (Tregs) and Immune Checkpoints

Enhancing Treg function or targeting immune checkpoints (including programmed death-1 [PD-1] and cytotoxic T-lymphocyte-associated protein 4) are innovative strategies for modulating immune tolerance in autoimmune diseases. Several experimental therapies aim to restore immune balance by promoting Treg stability and suppressing autoreactive T cells [46]. PD-1 is a prototypical immune checkpoint that mediates T cell suppression upon binding to its ligand, PD-L1. Several studies have indicated that PD-1 is associated with the pathogenesis of RA. In RA patients, increased PD-1 expression on T cells has been associated with elevated disease activity, indicating its potential as both a biomarker and a therapeutic target [47]. While monoclonal antibodies targeting the PD-1/PD-L1 axis have demonstrated significant success in oncology by enhancing antitumor immunity, their use is frequently accompanied by immune-related adverse events, including rheumatic manifestations such as inflammatory arthritis. This has led to the investigation of reverse strategies—namely, the agonistic engagement of PD-1—as a means to suppress autoreactive immune activity in autoimmune diseases such as RA.

Perisolimab, a humanized agonistic monoclonal antibody directed against PD-1, has been developed to exploit this immunoregulatory mechanism [47]. In a Phase IIa randomized, double-blind, placebo-controlled trial, adult patients with moderate-to-severe RA refractory to conventional or biologic/targeted synthetic DMARDs were assigned in a 2:1:1 ratio to receive 700 mg peresolimab, 300 mg peresolimab, or a placebo intravenously every 4 weeks. The primary endpoint was the change in DAS28-CRP from baseline to week 12, and this endpoint was met, i.e., the 700 mg group achieved a significantly greater improvement (least-squares mean change of −2.09 ± 0.18) versus placebo (−0.99 ± 0.26) (difference: −1.09; *p* < 0.001). Secondary endpoints included ACR20, ACR50, and ACR70 response rates. The 700 mg peresolimab group showed significantly higher ACR20 responses, while improvements in ACR50 and ACR70 were not statistically significant. Importantly, DAS28-ESR was not analyzed in this trial. The incidence of adverse events was comparable across all treatment arms during the study period, although extended follow-up will be necessary to assess long-term risks, including any potential influence on cancer susceptibility [9].

These findings suggest that PD-1 agonism may attenuate autoimmune pathology in RA, but further refinement is needed to enhance therapeutic efficacy, particularly in achieving higher-level clinical responses. Ongoing mechanistic investigations into the impact of PD-1 engagement on autoreactive T cells and autoantibody-producing B cells in RA may help to inform optimal dosing strategies and patient selection criteria for future trials.

### 2.5. Targeted B and T Cell Therapies

Telitacicept is a fusion protein that inhibits the TACI (transmembrane activator and CAML interactor) receptor expressed on B cells. This receptor mediates signaling through BAFF and APRIL, promoting B cell maturation and immunoglobulin production—processes often dysregulated in autoimmune diseases such as RA [48]. Clinical trials in SLE have validated the efficacy of Telitacicept, resulting in regulatory approval in China, and subsequent interest in its application to RA [49]. In a Phase III randomized, placebo-controlled clinical trial presented at the ACR Annual Meeting in November 2023 enrolled RA patients with an inadequate response to MTX. The primary endpoint was again the proportion of patients achieving an ACR20 response at week 24, while secondary endpoints included ACR50, ACR70, and changes in DAS28-ESR and other composite disease activity measures. Patients receiving Telitacicept achieved significantly greater improvements in ACR20, ACR50, and DAS28-ESR compared to placebo, with a safety profile comparable between treatment groups [10].

SM03, an anti-human CD22 monoclonal antibody, operates by enhancing SHP-1 signaling and dampening B cell receptor-driven NF-κB activation, thus limiting aberrant B cell responses [11]. In an initial Phase I open-label study, approximately half of the patients achieved ACR20 and DAS28 responses, with no severe adverse events reported [50]. A subsequent Phase II randomized trial in RA patients receiving background MTX therapy revealed that SM03-treated groups had significantly improved ACR20 responses at 24 weeks compared to the placebo group. Again, adverse event rates were similar across all arms [12]. A Phase III trial is currently underway to further define its efficacy and long-term safety.

CD40 and its ligand CD40L are members of the TNF receptor superfamily, with expression found not only on a broad range of immune cells but also on stromal and structural cell types, including fibroblasts, epithelial cells, and endothelial cells. CD40L serves as a crucial co-stimulatory molecule that modulates immune responses through engagement with CD40-expressing target cells, thereby initiating a cascade of downstream pro-inflammatory signaling events [13]. Dysregulation of this axis has been implicated in the pathogenesis of several autoimmune conditions, including SLE, RA, and Sjögren’s disease. In RA, the overexpression of CD40L has been associated with heightened autoantibody production and the exacerbation of disease activity. Moreover, the intra-articular activation of CD40 on synovial cells has been linked to the upregulation of inflammatory cytokines and mediators that directly contribute to cartilage degradation and bone erosion [14]. These findings have led to the hypothesis that the targeted inhibition of the CD40–CD40L interaction may represent a promising therapeutic avenue for autoimmune diseases [15]. Therapeutic agents designed to interrupt this pathway have demonstrated preliminary efficacy in both RA and other autoimmune conditions [16,17]. However, early clinical trials involving anti-CD40L humanized IgG1 monoclonal antibodies were prematurely terminated due to a high incidence of thromboembolic complications [18]. Subsequent mechanistic investigations revealed that the Fc region of the anti-CD40L antibody facilitated immune complex formation with recombinant CD40L, which then bound FcγRIIa (CD32a) on activated platelets, inducing platelet aggregation and thrombosis [19]. This FcγRIIa-mediated effect was not predicted in preclinical animal studies, as murine platelets lack expression of this receptor, underscoring the limitations of conventional animal models in safety profiling for biologic agents [20].

These findings have catalyzed the development of modified therapeutic strategies that preserve the immunomodulatory benefits of CD40–CD40L inhibition while minimizing thrombotic risk, including Fc silent antibody formats and alternative targeting mechanisms.

Dazodalibep (VIB4920), a recombinant fusion protein designed to inhibit the CD40–CD40L interaction, is currently under investigation as a novel therapeutic agent for RA. This agent selectively modulates T cell-dependent B cell activation, a critical pathway implicated in RA pathogenesis. In a Phase Ib randomized, placebo-controlled clinical trial involving patients with moderate-to-severe RA refractory to MTX or other DMARDs, multiple doses of dazodalibep were administered over a 12-week period. The trial demonstrated statistically significant improvements in disease activity, as measured by DAS28-CRP, in the higher dose cohorts when compared to the placebo cohort. Remarkably, clinical responses were sustained for at least three months following the final dose, despite subsequent transitions to other conventional therapies in many participants. The safety profile of dazodalibep was favorable, with adverse events such as diarrhea, upper respiratory tract infections, and urinary tract infections reported at comparable frequencies across treatment arms [21]. These findings highlight the therapeutic promise of targeting the CD40–CD40L co-stimulatory axis, supporting the continued clinical development of dazodalibep in RA.

OX40 (also known as CD134) and its ligand OX40L (CD252) are key members of the TNF receptor superfamily and play critical roles in regulating immune responses [51]. OX40 is expressed on activated T cells and serves as a co-stimulatory receptor during T cell activation [52]. In rheumatic diseases, OX40/OX40L signaling contributes to chronic inflammation and immune dysregulation—the hallmark features of conditions, such as RA, SLE, and other autoimmune disorders [53,54].

The OX40–OX40L signaling axis plays a crucial role in the amplification and persistence of effector T cell responses, particularly within CD4^+^ and CD8^+^ T cell populations. In the context of autoimmune pathology, the aberrant activation of this pathway contributes to the survival and expansion of autoreactive T cells, which can mediate sustained immune-mediated tissue damage [55]. Specifically, in diseases such as RA and SLE, OX40 signaling enhances the activity of pro-inflammatory T-helper subsets, including Th1 and Th17 cells, thereby perpetuating synovial inflammation and joint destruction. Additionally, this co-stimulatory interaction facilitates the T cell–B cell crosstalk, promoting the generation of pathogenic autoantibodies, a hallmark of RA progression. Experimental data have shown that OX40 signaling may impede Treg function and hinder peripheral Treg differentiation, further disrupting immune tolerance. These findings support the rationale for targeting the OX40/OX40L axis as a therapeutic intervention in autoimmune diseases [22]. Elevated OX40 expression has been detected in CD4^+^ T cells derived from both the peripheral circulation and inflamed synovial fluid of RA patients, with expression levels correlating positively with clinical disease activity [23]. Correspondingly, the increased expression of OX40 on T cells, and OX40L on B cells and macrophages, has been observed in RA synovial tissue, implicating this pathway in local immune dysregulation [24].

The therapeutic potential of OX40/OX40L blockade has been demonstrated in preclinical models, where monoclonal antibody inhibition attenuated inflammatory arthritis in various murine studies [25]. Several antagonistic antibodies—rocatinlimab, amlitelimab, and telazorlimab—have entered clinical development for atopic dermatitis, demonstrating early evidence of efficacy and safety [26,27,28,29].

### 2.6. Spleen Tyrosine Kinase (SyK) Inhibitors

SyK is a cytoplasmic non-receptor tyrosine kinase that plays a pivotal role in the intracellular signaling cascades downstream of immune receptors, particularly within B cells, Fc receptors, and integrins. Through these pathways, SyK modulates the activation, proliferation, and cytokine release of multiple immune cell populations. In the setting of RA, aberrant SyK signaling has been linked to the increased production of pro-inflammatory cytokines and matrix metalloproteinases, especially in response to TNF, thereby contributing to joint destruction and disease progression [30].

Fostamatinib, an orally bioavailable SyK inhibitor initially developed under the compound name R788, has demonstrated clinical efficacy in immune-mediated disorders and received FDA approval for the treatment of chronic immune thrombocytopenia [56]. Its anti-inflammatory properties have also been explored in preclinical models of arthritis, where it was shown to prevent the onset and progression of inflammatory joint damage [57]. In a Phase II randomized controlled trial involving RA patients with inadequate responses to MTX, adjunctive fostamatinib therapy produced clinically meaningful improvements in disease activity metrics [30,31].

The further evaluation of fostamatinib’s therapeutic utility in RA was undertaken in two Asia-based Phase II trials, OSKIRA-Asia-1 and OSKIRA-Asia-1X. In OSKIRA-Asia-1, the primary endpoint was the proportion of patients achieving an ACR20 response at week 12. Secondary endpoints included ACR50, ACR70, changes from baseline in DAS28-CRP and DAS28-ESR, and safety assessments [32]. This 12-week, placebo-controlled study compared various dosages of fostamatinib to placebo in patients on stable background MTX. Significant improvements were observed in ACR20 and ACR50 at week 8, and ACR70 at week 12, particularly in the 100 mg and 150 mg dosing arms. These cohorts also demonstrated superior reductions in DAS28-CRP scores relative to placebo. However, fostamatinib treatment was associated with several adverse events, including gastrointestinal disturbances (diarrhea and nausea), hypertension, elevated ALT levels, neutropenia, dizziness, headache, and upper respiratory tract infections.

The OSKIRA-Asia-1X trial, a long-term extension of the initial study, continued to monitor patients receiving 100 mg of fostamatinib. Common adverse effects in this extension study included nasopharyngitis, neutropenia, and sustained elevations in blood pressure [32]. Notably, a recent meta-analysis indicated that fostamatinib use was not associated with an increased risk of malignancy, although further large-scale observational studies are warranted to corroborate this finding [58]. With accumulating evidence on its immunomodulatory efficacy and manageable safety profile, fostamatinib represents a promising candidate for continued investigation in RA, potentially in head-to-head studies against established targeted therapies.

### 2.7. Anti-Granulocyte–Monocyte Colony-Stimulating Factor (GM-CSF) Antibodies

GM-CSF plays a critical role in the differentiation and activation of hematopoietic cells, particularly granulocytes and monocytes, and is implicated in the immune system dysregulation observed in inflammatory and autoimmune diseases. Elevated GM-CSF levels in inflamed tissues contribute to RA pathogenesis. Multiple anti-GM-CSF antibodies have been investigated for RA treatment. Mavrilimumab, an anti-GM-CSF receptor antibody, has demonstrated promising results across three pooled clinical trials, showing significant reductions in DAS28-CRP, ACR20, ACR50, and ACR70 scores compared to placebo after 12 weeks of therapy. No significant adverse events were observed [33]. However, despite these promising initial studies, no further trials have been conducted in recent years. Namilumab, an antibody targeting GM-CSF, was evaluated in a Phase II placebo-controlled study in patients with RA receiving MTX therapy. The study reported a significant improvement in DAS28-CRP scores in the high-dose namilumab groups than in the placebo group as early as week 2. Additionally, high ACR50 response rates were observed in the namilumab group at week 12. The overall safety profile was favorable, with minimal serious adverse events [34]. Otilimab is another anti-GM-CSF antibody that has advanced further in clinical studies. Initial Phase I and II studies demonstrated positive efficacy and safety profiles. The Phase III ContRAst 2 trial compared otilimab with the existing RA therapies tofacitinib and sarilumab. Although otilimab showed superior efficacy compared with that of placebo and maintained a positive safety profile, its efficacy was inferior to that of tofacitinib across multiple efficacy endpoints [35]. Additionally, in the ContRAst 3 trial, otilimab did not demonstrate significant efficacy differences compared to placebo, whereas sarilumab was superior to otilimab in ACR20 responses. Further large-scale clinical trials are required to determine their therapeutic potential in RA treatment.

### 2.8. Nuclear Factor-Kappa B (NF-κB) Inhibitor

Iguratimod is a csDMARD approved in Japan since 2012 for the treatment of RA. It suppresses B cell activity by inhibiting pro-inflammatory cytokines such as TNF-α, IL-1β, IL-6, IL-8, and IL-17, and downregulates NF-κB signaling, thereby reducing immunoglobulin production. By disrupting NF-κB-dependent signaling cascades, iguratimod effectively downregulates pro-inflammatory cytokine production and attenuates immune-mediated tissue damage [59]. Despite its widespread use in Japan, iguratimod is not currently included in international guidelines due to a lack of large-scale evidence outside East Asia. However, recent studies demonstrating its effects on lymphocytes, synovial fibroblasts, and clinical efficacy in real-world settings have renewed interest in its therapeutic potential [60].

Multiple clinical trials have demonstrated that iguratimod, either as monotherapy or in combination with MTX, confers superior clinical efficacy compared to MTX alone. A long-term comparative study found that the combination of iguratimod and MTX yielded significantly higher ACR20 response rates at week 52 than MTX monotherapy, with a favorable safety profile and no marked increase in adverse events [36]. The SMILE study, a multicenter, randomized, parallel-controlled trial, further substantiated these findings by showing that both iguratimod monotherapy and iguratimod plus MTX achieved superior clinical responses compared to MTX alone, while the incidence of adverse events remained comparable across all treatment arms over the 52-week study period [37]. In addition to efficacy, safety comparisons between iguratimod and other conventional DMARDs have yielded promising results. One study reported that iguratimod combined with MTX was better tolerated than the leflunomide–MTX combination, while demonstrating equivalent therapeutic efficacy [36]. Given these findings, and the ongoing accumulation of real-world safety and effectiveness data, iguratimod is increasingly being recognized as a viable alternative or adjunct to traditional DMARD regimens. Its encouraging safety profile and comparable or superior efficacy suggest that iguratimod may have potential for regulatory approval and broader use in regions beyond East Asia.

## 3. Future Perspectives

The identification of novel molecular targets continues to expand therapeutic options for RA. Advances in precision medicine, including biomarker-driven therapies and gene-editing technologies, hold promise for more personalized and effective treatments. Ongoing research on the interplay among genetic factors, microbiome influences, and immune dysregulation will further shape the future of RA management.

## 4. Conclusions

The discovery of emerging molecular targets presents new opportunities for the development of novel therapies for rheumatic diseases. Targeting key signaling pathways, such as BTK, NLRP3, and immune checkpoints, offers promising strategies for improving patient outcomes. As research progresses, the integration of novel immunomodulatory strategies with precision medicine approaches is expected to transform the treatment paradigm for autoimmune rheumatic conditions.

## Figures and Tables

**Table 1:** Summary of novel molecular therapeutic targets in rheumatoid arthritis.

Target	Drug(s)	Mechanism	Clinical Status	References
Bruton’s Tyrosine Kinase (BTK)	Fenebrutinib, Evobrutinib	B cell receptor signaling inhibition, reducing autoantibody production	Phase II trials showed the efficacy Fenebrutinib to be similar to that of Adalimumab; Evobrutinib showed safety but lacked efficacy.	[4,5]
Mitochondrial Complex I	MBS2320 (Leramistat)	Selective osteoclast inhibition and anti-inflammatory action	Phase II trial demonstrated significant ACR20 and DAS28-CRP improvement.	[6]
NLRP3 Inflammasome	Dapansutrile	Suppresses IL-1β and IL-18 production via inflammasome inhibition	Upregulation in RA confirmed; early-stage therapeutic potential	[7,8]
PD-1 Immune Checkpoint	Peresolimab	Restores T cell tolerance and suppresses autoimmune inflammation	Phase II trial showed DAS28-CRP and ACR20 improvement in the high-dose group.	[9]
B/T Cell Co-stimulation	Telitacicept	Blocks TACI-mediated B cell maturation and immunoglobulin production	Phase III trial showed significant ACR and DAS28-ESR improvements.	[10]
B Cell CD22	SM03	Modulates SHP-1 and inhibits BCR/NF-κB signaling	Phase I/II trials demonstrated safety and clinical efficacy.	[11,12]
CD40/CD40L	KLP-404, VIB4920	Inhibits B and T cell co-stimulatory signaling to reduce inflammation	Modified Fc structure reduces thromboembolic risks; early trials positive	[13,14,15,16,17,18,19,20,21]
OX40/OX40L	Rocatinlimab, Amlitelimab, Telazorlimab	Suppresses effector T cell activation and enhances Treg stability	Trials ongoing in dermatitis; preclinical studies support RA application.	[22,23,24,25,26,27,28,29]
Spleen Tyrosine Kinase (SyK)	Fostamatinib	Blocks immune activation via B cell receptor and Fc receptor pathways	Phase II trials (OSKIRA) confirmed efficacy; manageable safety profile	[30,31,32]
GM-CSF	Mavrilimumab, Namilumab, Otilimab	Neutralizes granulocyte/monocyte stimulation in inflamed joints	Mixed outcomes: early agents promising; Otilimab underperforms in Phase III.	[33,34,35]
NF-κB	Iguratimod	Blocks nuclear translocation of NF-κB, dampening inflammation	Widely used in Asia; effective as monotherapy or with MTX; superior to MTX alone	[36,37]
